# The Grafting of Multifunctional Antithrombogenic Chemical Networks on Polyurethane Intravascular Catheters

**DOI:** 10.3390/polym12051131

**Published:** 2020-05-15

**Authors:** Yael Roth, Dan Y. Lewitus

**Affiliations:** Department of Plastics and Polymer Engineering, Shenkar Engineering, Design, Art, Ramat Gan 52526, Israel; yaelr@shenkar.ac.il

**Keywords:** polyurethane, thiol-ene click reaction, antithrobmogenic, catheter, in vivo

## Abstract

Intravascular catheters (IVCs) and other medical tubing are commonly made of polymeric materials such as polyurethane (PU). Polymers tend to be fouled by surface absorption of proteins and platelets, often resulting in the development of bacterial infections and thrombosis during catheterization, which can lead to embolism and death. Existing solutions to fouling are based on coating the IVCs with hydrophilic, anti-thrombogenic, or antimicrobial materials. However, the delamination of the coatings themselves is associated with significant morbidity, as reported by the United States Food and Drug Administration (FDA). We developed a lubricious, antimicrobial, and antithrombogenic coating complex, which can be covalently attached to the surface of industrial PU catheters. The coating complex is pre-synthesized and comprises 2-methacryloyloxyethyl phosphorylcholine (MPC) as an antifouling agent, covalently attached to branched polyethyleneimine (bPEI) as a lubricating agent. The two-step coating procedure involves PU-amine surface activation using a diisocyanate, followed by chemical grafting of the bPEI-S-MPC complex. Compared with neat PU, the coating was found to reduce the coefficient of friction of the IVC surface by 30% and the hemolysis ratio by more than 50%. Moreover, the coating exhibited a significant antimicrobial activity under JIS Z2801:2000 standard compared with neat PU. Finally, in in-vivo acute rabbit model studies, the coating exhibited significant antithrombogenic properties, reducing the thrombogenic potential to a score of 1.3 on coated surfaces compared with 3.3 on uncoated surfaces. The materials and process developed could confer lubricious, antithrombogenic, and antimicrobial properties on pre-existing PU-based catheters.

## 1. Introduction

Intravascular devices, such as catheters, are crucial to modern-day medical practice. Such medical devices are used to administer parenteral nutritional fluids, drugs, and intravenous fluids, and to monitor the hemodynamic status of critically ill patients. Thus, the interactions of their surfaces with biological systems are of major importance [1]. Catheters and medical tubing are commonly made of synthetic materials, including silicones, polyurethanes (PU), polyamides, polyolefins, and polyvinylchloride. PU has been extensively used as a biomaterial in medical devices owing to its flexibility, elasticity, and excellent abrasion resistance [2]. PU is known to be more biocompatible and blood compatible than other synthetic polymers and is used in a wide range of applications, from catheters to total artificial hearts [3].

Several risks are associated with the use of synthetic polymers in intravascular devices. First, fouling occurs, which may in turn lead to thrombosis or bacterial infection [1,2,4,5]. Central venous catheters and peripherally inserted central catheters have serious potential to cause life-threatening sepsis, and catheter infection rates are 5.3 per 1000 catheter days [6,7]. With most catheters, soon after their insertion, a fibrin sheath is formed around the catheter. This fibrin sheath, in the case of long-term catheterization or poor handling, can lead to acute thrombosis [1]. Of all catheters placed, between 42% and 100% develop fibrin sheaths, and between 20% to 40% develop pericatheter thrombus [1]. Once a pericatheter thrombus or fibrin sheath occurs, the patient is predisposed to infection. Similarly, a pericatheter infection may increase the risk of thrombosis [2,3] Furthermore, the thrombus may detach from the surface and drift away, resulting in thromboembolism, which increases patients’ mortality [4]. Second, the insertion of the catheter through mucous membranes or vascular surfaces inevitably results in irritation of the area in immediate contact with the device. Additional damage and appreciable discomfort to the patient are caused by the high coefficient of friction (COF) between the blood vessel and the catheter surface, as well as by any subsequent movement by the patient [5]. Conventional medical practices aimed at preventing thrombosis include the direct administration of anticoagulant agents such as heparin to patients who are exposed to blood-contacting medical devices. Although this approach is effective in reducing blood coagulation, it presents the undesirable risk of uncontrollable patient bleeding [6].

Thus, to alleviate the aforementioned risks, multifunctional coatings for intravascular catheters have been developed over the past 6–7 decades [7]. Coatings found in medical use today have desirable properties such as lubricity and antithrombogenicity. The first generation of coatings was focused on hydrophilic materials, such as hydrogels [7]. Hydrophilic lubricious coatings reduce the potential for various infections by significantly reducing protein adherence to the substrate. However, lubrication itself does not ensure the prevention of other undesired biological reactions [6,8,9]. Further developments involved the integration of specific chemical species and charges within the coating, such as anti-bacterial and anti-thrombogenic molecules [10,11]. These coatings not only provide lubricity and biocompatibility but also serve as drug reservoirs for local drug delivery [12,13].

Recently, some commercial coatings were found to increase the risk of embolism, and FDA reports from the past decade show that existing coatings can detach from the surface, resulting in embolism and even death [14]. Since 1 January 2010, there have been 11 recalls of products from various manufacturers associated with these coatings peeling or flaking off medical devices. In addition, since 1 January 2014, the FDA has received approximately 500 medical device reports (MDRs) describing separation of hydrophilic and/or hydrophobic coatings on medical devices such as guidewires and catheters. Serious injuries associated with the peeling of coatings reported in MDRs included the persistence of coating fragments in patients, requiring surgical intervention to mitigate the consequences, adverse tissue reactions, and thrombosis [14], These findings suggest that future coatings must have abrasion resistance in addition to the aforementioned properties. 

Whereas hydrophilic polymers such as polyethylene glycol, polyvinylpyrolidone, PU, polyacrylic acid, and polysaccharides can form a hydration layer via hydrogen bonds, zwitterions form a hydration layer via electrostatic interactions [15,16,17]. Zwitterions are capable of binding a significant number of water molecules and are therefore potentially excellent candidates for low fouling materials [15,18,19]. Moreover, they have been shown to be non-thrombogenic [15,16,17]. MPC-based polymers have been shown to significantly reduce protein adsorption compared with relevant controls and have been widely used in various applications [10,20,21]. Antifouling and antithrombogenic coatings have been developed based on phosphorylcholine functioning [19,22,23,24].

Chemical modifications of PU surfaces can be used to incorporate the desired functional groups [22,23]. This may allow a covalent linkage to be generated between an antithrombogenic agent and the surface of an existing intravascular medical device, which in turn may alleviate detachment and delamination. Isocyanate can react with the secondary amine in an urethane bond to yield a substituted urea bond, also called allophanate [24,25,26]. Tan et al. [17] grafted branched polyethyleneimine (bPEI) onto a PU in solution via a diisocyanate mediator, followed by further grafting of PC, to provide an antithrombogenic PU complex prepared via solvent casting. However, this method is not applicable to commercial PU catheters. 

In this study, a new method was developed to modify existing PU surfaces and address the aforementioned acute requirements, while maintaining the material and design of commercial PU devices. The grafting procedure consists of two identical steps, comprising the functionalization of the PU surface using a diisocyanate mediator and the conjugation of a pre-synthesized lubricious, antimicrobial, and antithrombogenic coating complex consisting of MPC substance as the antithrombogenic agent, covalently attached to thiolated bPEI (bPEI-SH). 

## 2. Experimental

### 2.1. Materials

All chemicals were purchased and used as received. Polyethyleneimine (PEI branched, *M*_w_ 800 by light scattering), ethylene sulfide (ES, 98%), 2-methacryloyloxyethyl phosphorylcholine (MPC, 97%), 2,2-dimethoxy-2-phenylacetophenone (DMPA 99%), fluorescein-O-methacrylate (95%), p-toluene sulfonyl isocyanate (TSC, 96%), hexamethylene diisocyanate (HDI ≥ 99%), dibutyltin dilaurate (DBTDL, 95%), were all purchased from Sigma-Aldrich Nes Tiona, Israel). 5,5′-dithiobis(2-nitrobenzoic acid) (DTNB, 97%), and *N*-acetyl-l-cysteine (C_5_H_9_NO_3_S, 98+%) were all purchased from Alfa Aesar (Yehud, Israel). Toluene AR-b, ethanol absolute (dehydrated) AR-b, methanol AR-b, diethylether AR were all purchased from Bio-Lab (Jerusalem, Israel). Medical grade PU was generously donated by Lubrizol (Bowling Green, OH, USA).

### 2.2. Synthesis of the Lubricious, Antimicrobial, and Antithrombogenic Grafting Complex

bPEI-SH was synthesized through ring opening of ES [27]. Six grams of bPEI were dissolved in a 9:1 mixture of toluene: ethanol solution in a 100 mL round flask. The solution was refluxed under a nitrogen atmosphere for 15 min, then 400 µL ES was added dropwise over 1 min. The reaction was washed by refluxing for 2 h, followed by the removal of the solvents by evaporation under reduced pressure. The thiolation of bPEI was analyzed using infrared (IR) spectroscopy, ultraviolet-visible light (UV–VIS) spectroscopy, and fluorescence microscopy.

bPEI-S-MPC was synthesized through the thiol-ene click reaction; 800 mg of MPC and 20 mg of DMPA were dissolved in 4.5 mL of methanol and added to 2 g of bPEI-SH. The mixture was irradiated under UV light (20-watt, 365 nm, Analytik Jena, Beverly, MA, USA) for 40 min. The solvent was removed by rotary evaporation under reduced pressure. The product was analyzed using IR spectroscopy and elemental analysis.

### 2.3. IR Spectroscopy

Absorption spectra were obtained by Fourier transform infrared spectroscopy (Bruker, Ettlingen, Germany) using the attenuated total reflection method (ATR-FTIR). The tested samples were bPEI, bPEI-SH, bPEI-S-MPC. Using OPUS software (Bruker, Ettlingen, Germany), the surface of each sample was scanned 100 times, and the infrared spectra were obtained. The average resolution of the measurement was adjusted to 2 cm^−1^.

### 2.4. UV–VIS Spectroscopy

DTNB, also called Elman’s reagent, was used for the detection of free thiol groups using UV–VIS spectroscopy. A reaction buffer solution was prepared using 0.1 M sodium phosphate (pH 8) and 1 mM ethylenediaminetetraacetic acid. Elman’s reagent solution was prepared by dissolving 4 mg Elman’s reagent in 1 mL of the reaction buffer. 

Then, 4.6 mg/mL bPEI-SH in distilled water was added to 50 µL Elman’s reagent solution, followed by the addition of 250 µL of reaction buffer. The mixture was agitated for 15 min at 150 rpm in the dark for the measurements. As a reference, bPEI was tested following the same procedure. The absorbance was detected using a UV–VIS spectrophotometer (UV-1650PC, Shimadzu Corporation, Kyoto, Japan).

### 2.5. Fluorescent Probe

PEI-S-FOM was synthesized through the thiol-ene click reaction; 200 mg of fluorescein-o-methacrylate (FOM) and 10 mg DMPA were added to 2 mL methanol containing 1 g PEI-SH. The reaction was irradiated under UV light (20-watt, 365 nm, Analytik Jena) for 1 h, followed by washing with an excess of methanol. The product was analyzed under a fluorescence microscope (CKX53, U-MSWG filter, Olympus, Tokyo, Japan).

### 2.6. Elemental Analysis

Atomic percentages of the coating complex were obtained by elemental analysis. The tested samples were bPEI, bPEI-SH, bPEI-S-MPC. The C, N, H, and O percentages were measured using a Flash 2000 CHN-O Elemental Analyzer (Thermo Fisher Scientific, Bedford, MA, USA). This system uses a simultaneous flash combustion method (950–1060 °C) for C, H, N, and pyrolysis of oxygen to convert the sample elements to simple gases. The gases are detected as a function of their thermal conductivity. The S and P percentages were determined using microwave-induced oxygen combustion (Anton Paar, Graz, Austria) for the decomposition of organic samples and ion chromatography analysis using a Dionex IC system (Thermo Fisher Scientific, Bedford, MA, USA).

### 2.7. Preparation of PU Surfaces`

The samples to be coated were prepared by solvent casting onto glass petri dishes. PU resins were dissolved in tetrahydrofuran at a concentration of 2% *w*/*v*. Air plasma was applied to glass petri dishes (90 mm in diameter) for 5 min, followed by casting of 15 mL of the PU solution. After slow evaporation of the solvent in a chemical fume hood, the casted dishes were dried overnight at 55 °C and maintained under vacuum. 

### 2.8. Grafting on PU Surfaces

PU-HDI was synthesized as follows. The PU surface was soaked in toluene and 0.25% *v/v* DBTDL. The reaction was conducted for 60 min at 70 °C under orbital shaking at 50 rpm. The modified PU surface was analyzed using ATR-FTIR spectrophotometry and X-ray photoelectron spectroscopy (XPS).

PU-HDI-bPEI-S-MPC was synthesized by the same reaction procedure; 1 g of either bPEI, bPEI-SH, or bPEI-S-MPC and 0.25% *v/v* DBTDL were added to toluene and the solution was spread over a functionalized surface. The reaction was conducted for 60 min at 70 °C under orbital shaking at 50 rpm. The coated surfaces were washed twice with an excess of toluene for 15 min and twice with methanol. The coated surfaces were analyzed using XPS and elemental analysis. The COF of coated PU was measured in distilled water. The hemolysis ratio of the surfaces was calculated, and antimicrobial and antithrombogenic tests were performed.

### 2.9. X-ray Photoelectron Spectroscopy 

XPS measurements were performed on a Kratos Axis Ultra X-ray photoelectron spectrometer (Karatos Analytical Ltd., Manchester, UK). The tested samples were PU neat surface, PU-HDI and PU-HDI-bPEI. The samples size was 5 × 5 mm^2^. High-resolution XPS spectra were acquired with a monochromatic Al Ka X-ray radiation source (1486.6 eV) with 90° takeoff angle (normal to the analyzer). The pressure in the chamber was 2·10-9 Torr. The high-resolution XPS spectra were collected with pass energy 20 eV and step 0.1 eV. Data analyses were performed using Casa XPS (Casa Software Ltd., Teignmouth, UK) and the Vision data processing program (Kratos Analytical Ltd., Manchester, UK).

### 2.10. Coefficient of Friction Measurements

The COF of coated surfaces was measured in distilled water. For this purpose, a bath was constructed to fit the standard apparatus to perform a standard COF test according to ASTM 1894 (Supplementary Figure S1). The bath was filled with 30 mL distilled water and each sample was tested for 25 cycles. The remained liquid was collected in an empty vial to evaluate whether particulates had been formed. The collected water was examined using a particle size analyzer (Malvern Zetasizer nano ZS, Malvern, UK) and by energy dispersive X-ray spectroscopy after evaporation of the liquid. Data were calculated (average with standard deviation), and presented using GraphPad^®^ PRISM 8 (GraphPad, San Diego, CA, USA). Multiple T-test were used for statistical analyses.

### 2.11. Ex-Vivo Evaluations 

Antimicrobial activity of the coating was evaluated according to the JIS Z2801: 2000 test. Coated and uncoated PU samples films were cut using dermal biopsy punch (Albion) to a size of 5 mm in diameter. The tested samples were solvent cast PU, PU-HDI, PU-HDI-bPEI, PU-HDI-bPEI-SH, PU-HDI-bPEI-S-MPC (Supplementary Figure S11). The samples were incubated with Escherichia coli bacteria for 24 h at 37 °C in a humid atmosphere. Then, the samples were sonicated to detach all bacteria on the surface. The sonicated liquid was cultured on blood-agar petri dishes, followed by another overnight incubation at 37 °C in a humid atmosphere. The antimicrobial activity of each sample was determined by the number of colony forming units (CFU) developed over the culture petri dishes.

Hemolysis testing was performed as described in the study of Wang et al. [28]. Each sample (5 mm in diameter) was soaked in 160 µL phosphate-buffered saline (PBS) at 37 °C for 30 min. Then, 510 µL fresh blood from healthy pigs (containing 6% *v*/*v* of 20 mg/mL potassium oxalate) was added to each sample following incubation at 37 °C for 60 min, and samples were centrifuged at 1350 rpm for 5 min. The absorbance of the supernatant solution was measured using a plate reader at a wavelength of 545 nm. The hemolytic ratio (HR%) was calculated using the following equation: (HR%) = ((As − An)/(Ap − An)) × 100, where A_s_ is the obtained absorbance of the tested sample, A_nc_ and A_pc_ are the absorbance of the negative control (0.02% *v*/*v* of diluted blood in PBS) and positive control (0.02% *v*/*v* of diluted blood in DI water), respectively. Data were calculated (average with standard deviation), and presented using GraphPad^®^ PRISM 8. Multiple T-test were used for statistical analyses.

### 2.12. In-Vivo Thrombogenicity Evaluation 

The thrombogenic potential of the coating was based on the Wessler stasis model in rabbits, performed in compliance with the Israel Animal Welfare Act and following approval by the Israel Board for Animal Experiments Ethics Committee (# IL-19-4-180). PU-coated nitinol wires (30 mm × 0.5 mm) were provided by Medibrane^TM^ (Rosh HaAyin, Israel). Three wires were grafted with bPEI-S-MPC according to the grafting procedures described in Section 2.8. A further three wires were used as received as a positive control. Thrombogenic potential was studied in white New Zealand White rabbits weighing 2.5 kg. Animals were anesthetized with intramuscular ketamine/xylazine, 35/5 mg/kg, respectively, at a dose volume of 0.3 mL/kg. Hair at the area of the femoral artery was removed, and the area was disinfected using Polydine solution. The animal was placed on a heating pad during the surgery. A skin incision of 2.0 cm was performed and the femoral artery was exposed. The femoral artery was raised using a surgical thread and clipped in the upper part before the thread to stop the blood flow. A small incision was performed using scissors (Supplementary Figure S13). The tested article was inserted through the cut towards the abdominal aorta using a cannula. The implant was left for 180 min, the duration of the study. At termination, animals were sacrificed using an overdose of intravenous pentobarbital (150 mg/kg). The segment of the aorta with the implant was closed from both sides and removed gently. Its contents were emptied into a Petri dish containing 50 mL 0.9% saline. The petri dish was photographed and the number of clots around each test article were counted. The thrombogenic potential was examined using an optical microscope as follows: 0, no clot; 1, few macroscopic standards of fibrin; 2, several small thrombi; 3, two or more large thrombi; 4, a single thrombus forming a cast of the isolated segment.

## 3. Results and Discussion

### 3.1. Synthesis of bPEI-SH

**Scheme 1:**
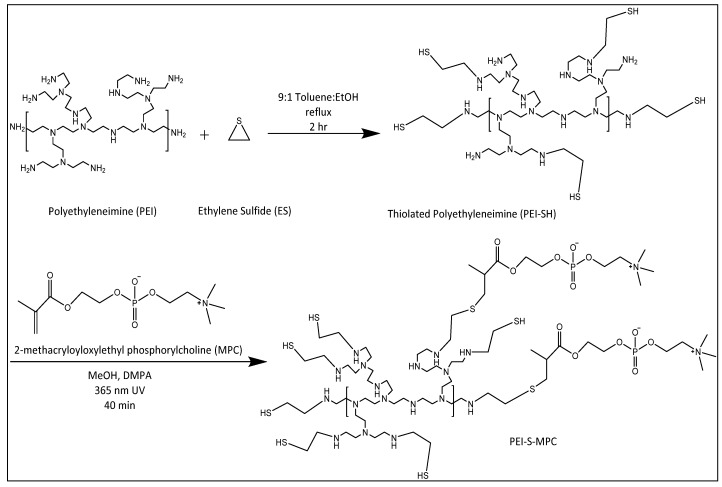
Production of the grafting complex.

Scheme 1 illustrates the pathway for the synthesis of the bPEI-S-MPC coating complex. The bPEI-SH was produced by reacting bPEI amines with ES. A pair of unconjugated electrons on the sulfur atom, along with the cyclic tension of a three-membered ring, makes the ES molecule highly reactive, with the amine acting as a nuclephile [29,30]. The attachment of the MPC to the formed thiols in the bPEI-SH was performed via the thiol-ene click reaction [31], resulting in the final bPEI-S-MPC coating complex. 

**Figure 1:**
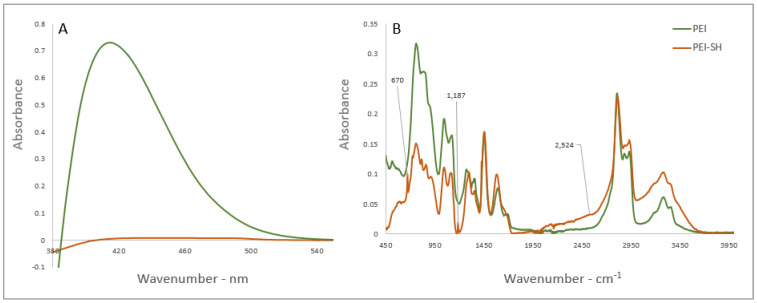
(**A**) UV–VIS spectra of TNB, the decomposition product of Elman’s reagent and; (**B**) ATR-FTIR spectra of bPEI-SH and bPEI neat.

IR spectrum of substances express the stretch and vibration of specific sets of chemical bonds from within a molecule [17]. The ATR-FTIR absorption spectra of bPEI-SH and untreated bPEI are shown in Figure 1B. The peaks at 670 cm^−1^ and 2524 cm^−1^ are at frequencies associated with thiol stretching [17] indicating the presence of thiols in bPEI. However, evidence of thiols in IR spectra can be misleading, as C–S–H and C–S stretching vibrations are associated with relatively weak absorptions in the IR spectrum [17]. Thus, UV–VIS analysis was performed to support the IR findings (Figure 1A), following the use of Ellman’s reagent for the detection of free thiol groups [17], Elman’s reagent (DTNB) reacts with a free sulfhydryl group to yield a disulfide molecule and 2-nitro-5-thiobezoic acid (TNB). Elevated absorption in the range UV–VIS is associated with the presence of TNB, which indicates the presence of thiol free groups in a tested sample. Moreover, the exposure of unmodified bPEI to Elman’s reagent solution did not result in the decomposition of the reagent. Both the ATR and the UV–VIS observations indicate the formation of bPEI-SH. 

### 3.2. Thiol-Ene Click for Conjugation of MPC

**Figure 2:**
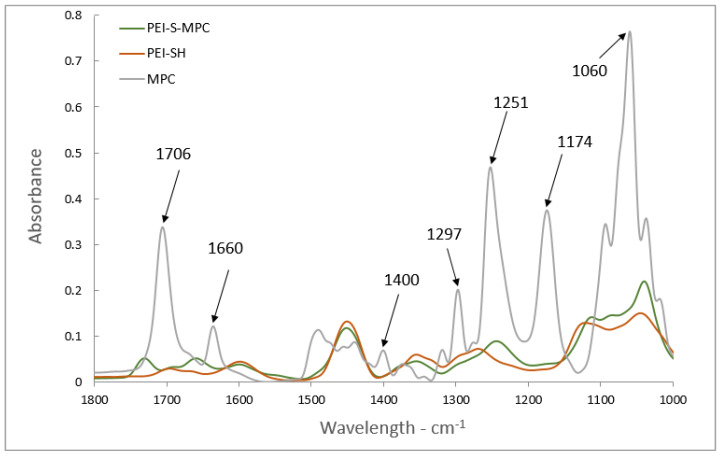
ATR-FTIR spectra of the coating complex (bPEI-S-MPC), bPEI-SH, and MPC reagent.

The IR spectra of the MPC reagent, bPEI-SH, and bPEI-S-MPC (prepared according to Scheme 1) are shown in Figure 2. The peaks at 1660 cm^−1^, 1174 cm^−1^, and 1060 cm^−1^ correspond to the C=C double bond, and those at 1400 cm^−1^ and 1297 cm^−1^ correspond to H-C=C bending; all these peaks originate from the methacrylate group of MPC. The double bond of MPC opens during the reaction, binding the MPC molecule to bPEI-SH via the thiol group. Thus, the alkene peaks were not present in the coating complex, bPEI-S-MPC. The peaks at 1706 cm^−1^ and 1251 cm^−1^ correspond to the carbonyl and phosphonate groups of MPC, respectively. These peaks were observed for bPEI-S-MPC, indicating the presence of MPCs in the end product. 

Elemental analysis was performed to confirm the ATR data and to evaluate the actual atomic ratio in each of the three compounds. The atomic distributions as percentiles are presented in Table 1. After the thiolation of bPEI, 2.6% of the detected atoms were found to be sulfur. Phosphorous was detected only in bPEI-S-MPC, with an atomic percentage of 2.1%. 

**Figure 3:**
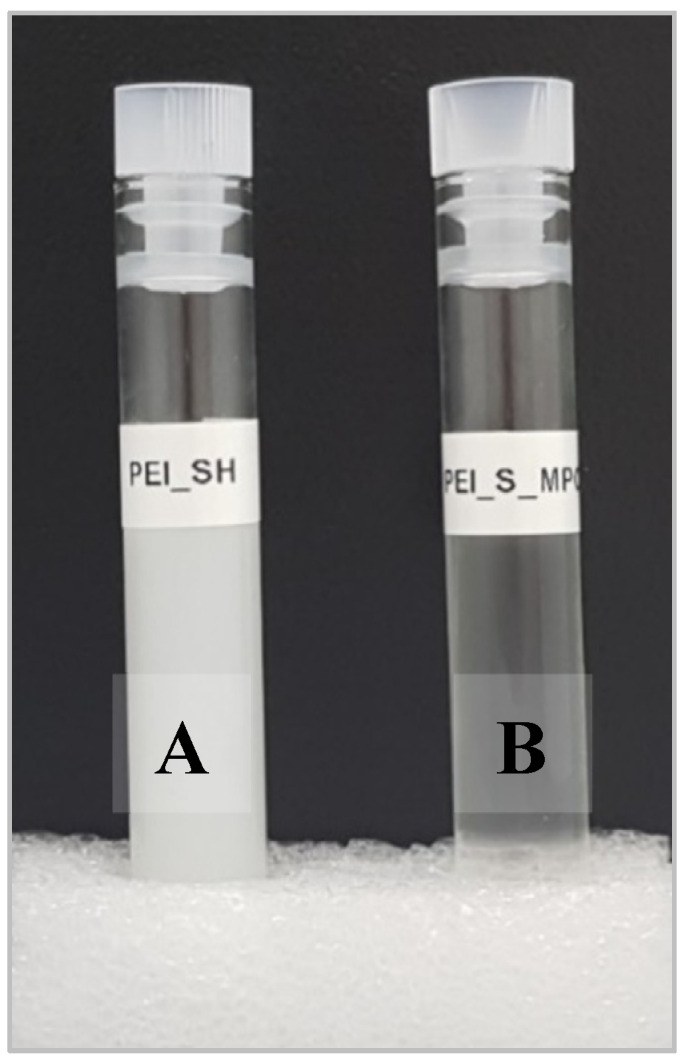
Water solubility of 60 mg/mL thiolated bPEI (bPEI-SH) (**A**) and the grafting complex (bPEI-S-MPC) (**B**).

Visually, there were no observable changes between bPEI-SH and bPEI-S-MPC. However, bPEI-S-MPC became soluble in water, whereas bPEI-SH was insoluble. As shown in Figure 3, the mixture of bPEI-SH in water was opaque, indicating its immiscibility in water, whereas the bPEI-S-MPC solution was transparent. This difference was ascribed to the addition of MPC onto bPEI-SH, which adds polar interactions and contributes to the solubility of bPEI-S-MPC in water. 

### 3.3. Functionalization of PU Surfaces Using Toluenesulfonyl Isocyanate

The reaction between an isocyanate functional group and the secondary nitrogen, which is present in a urethane linkage, results in the formation of a substituted urea linkage, also called allophanate (Supplementary Scheme S1). Toluenesulfonyl isocyanate is a monofunctional substance that was used to simplify the optimization of the reaction conditions. An optimization was made for the reaction parameters, including the reaction temperature, duration, and the necessity of a catalyst within the reaction. A semi-quantifying analysis was performed by peak integration (data shown in ESI). The ideal product was observed at a temperature of 70 °C for 60 min.

### 3.4. Isocyanate Functionalization

**Scheme 2:**
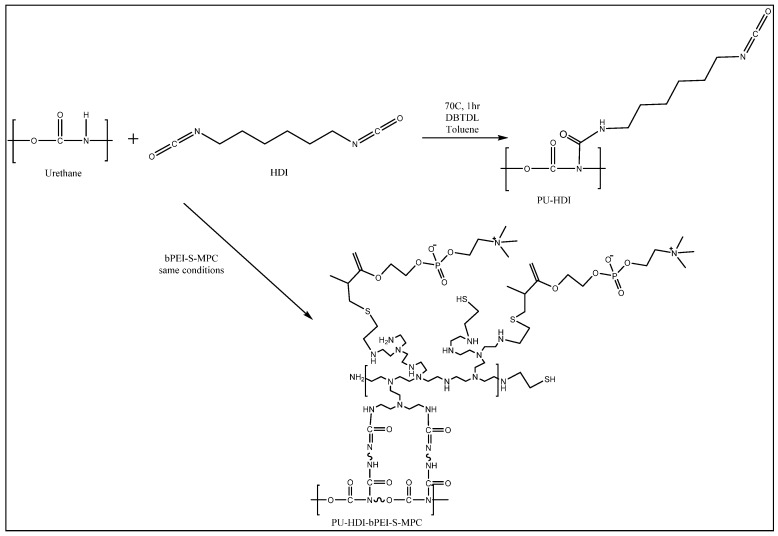
Suggested pathway for the application of the coating complex on PU solvent-casted surfaces using HDI as the coating mediator.

The use of diisocyanate as the coating mediator simplifies the application of the coating on PU surfaces [17]. As the coating complex, bPEI-S-MPC, consists of free amine end-groups, it can bind to free isocyanate groups similarly to urethane. Using diisocyanates, one end-group can be conjugated to the PU surface, while the other functional group is capable of binding bPEI-S-MPC. Thus, the application of the coating on an industrial PU catheter involves two identical steps, as shown in Scheme 2. 

**Figure 4:**
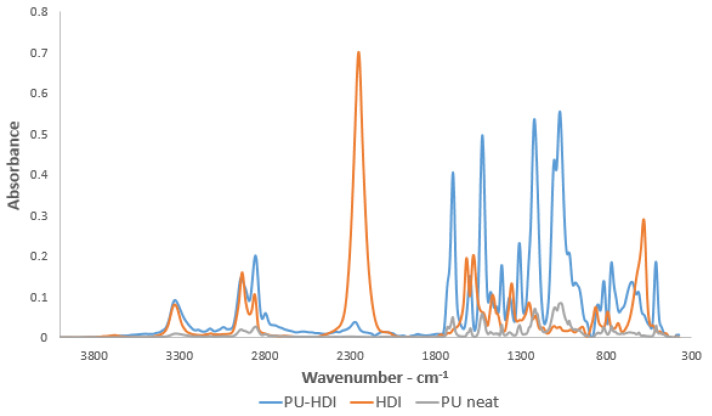
ATR-FTIR spectra of functionalized PU surface (PU-HDI); PU neat and HDI reagent.

Using ATR-FTIR analysis, improves the surface sensitivity of the FTIR technique, as the Sampling depth is in the order of microns,, thus indicates of chemical interactions on the surface of a tested substance [17,32,33]. HDI was grafted on the PU surface. An absorption peak was observed in the ATR-FTIR spectrum of the functionalized PU-HDI surface (Figure 4) at 2222 cm^−1^, indicating the presence of an isocyanate functional group on the surface after the reaction. 

### 3.5. The Preparation of PU-HDI-bPEI-S-MPC

**Scheme 3:**
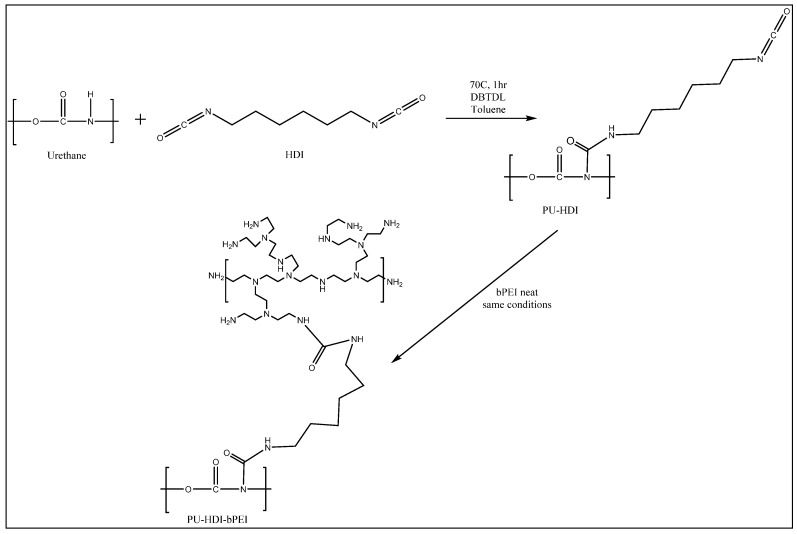
Suggested pathway for the application of bPEI to a PU surface through functionalization of the PU surface with HDI.

Prior to the application of the grafting complex on the functionalized PU surfaces, unmodified bPEI was grafted on PU-HDI. Scheme 3 illustrates the suggested pathway for the application of bPEI onto a PU surface.

XPS analysis was used as a complementary method to support ATR-FTIR findings. The results of the XPS analysis of PU surface, PU-HDI, and PU-HDI-bPEI are shown in Table 2. The unmodified PU surface was found to be rich in oxygen-containing fragments, indicating that the initial amount of nitrogen atoms was relatively low. However, the grafting of HDI and bPEI onto the surface resulted in a significant increase in nitrogen atoms. The O/N ratio was significantly reduced after grafting of HDI or bPEI. Moreover, the grafted surface with bPEI contained more than four times the number of nitrogen atoms compared with unmodified PU.

### 3.6. Mechanical Properties

The grafting of HDI onto the surface of PU, as well as the exposure of the substrate to reaction conditions, can potentially influence the mechanical properties of PU [17]. Mechanical properties were tested using a film tensile test for the unmodified PU surface, and for PU that had been exposed to the reaction conditions with or without the grafting of isocyanate. Supplementary Figures S8–S10 show the elastic modulus, strength, and elongation at break of samples, respectively. Neither the reaction conditions nor the grafting of HDI appeared to have a significant influence on the mechanical properties of the substrate.

### 3.7. Coefficient of Friction

**Figure 5:**
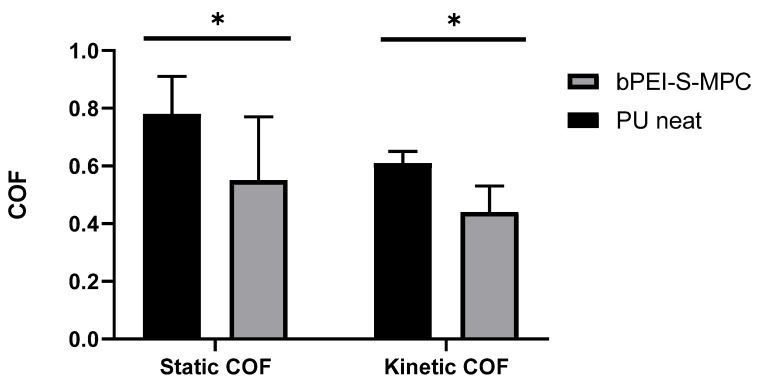
COF measurements for PU neat and bPEI-S-MPC. (*n* = 50, * denotes *p* < 0.01).

The COF values of bPEI-S-MPC-coated PU surfaces were measured for comparison with the neat PU surface. Figure 5 shows the average results for 50 cycles of the friction test (two samples of each type were tested for 25 cycles each), normalized to the neat PU value. The bPEI-S-MPC coating resulted in improved lubricity of the surface, reducing the COF of the surface by approximately 30%. 

### 3.8. Antimicrobial Activity

The JIS Z2801:2000 test evaluates the antimicrobial activity of a substance through culture of a known bacterial concentration per unit of surface area [34]. A series of samples were prepared for the JIS study to assess the various steps of the coating procedure, including a neat PU surface that had been exposed to the reaction conditions (PU), the PU grafter with HDI (PU-HDI) and application of bPEI (PU-HDI-bPEI), thiolated bPEI (PU-HDI-bPEI-SH), and the whole coating complex (PU-HDI-bPEI-S-MPC). A graphic illustration of the tested samples is shown in Supplementary Figure S11. 

Supplementary Figure S12 shows the blood-agar petri dishes at the end of the test. Table 3 summarizes the CFU count for each tested sample. The neat PU (exposed to reaction conditions only) exhibited a large accumulation of E. coli on the surface, with more than 200 CFU observed. A similar result was obtained for the PU surfaces that were grafted with HDI. The conjugation of bPEI on the functionalized PU-HDI was found to reduce the CFU value significantly to an average of 50. The presence of thiol groups, as well as MPC molecules, resulted in no bacteria at all on the blood-agar petri dishes, indicating that these surfaces possess significant antimicrobial activity [17,35].

### 3.9. Hemolysis Assay

A material is considered hemolytic if it causes fragmentation of red blood cells (RBCs) [28]. When a dose of blood is centrifuged, a separation of the blood components is observed as a function of weight. RBCs descend, as they are the heaviest component of blood, while the upper extract consists of the blood plasma. Fragments of damaged RBCs tend to mix with the plasma, and the upper layer of a hemolyzed portion of blood becomes reddish. Thus, the color change of the extract can be used to measure the degree of hemolysis [28,36]. 

**Figure 6:**
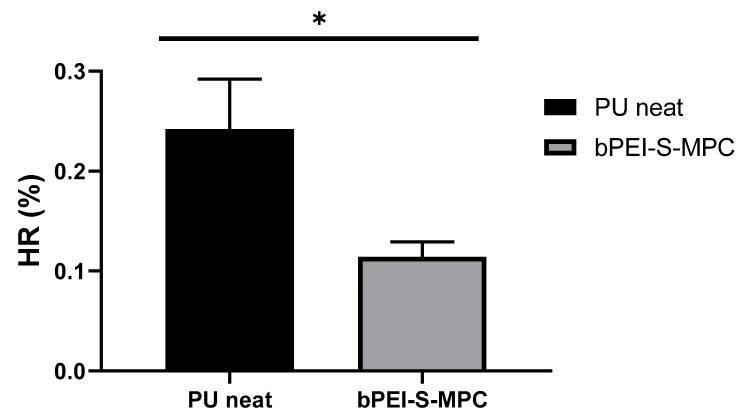
Hemolysis ratio of PU neat and bPEI-S-MPC (*n* = 5, * denotes *p* < 0.01).

The hemolysis ratio was calculated for unmodified PU (PU-neat) and PU-bPEI-S-MPC [28]. Distilled water (which is highly hemolytic) was used as the positive control, and PBS was used as the negative control. The HR% values of the tested samples are given in Figure 6, normalized to the PU hemolytic value. The bPEI-S-MPC coating complex was found to reduce the hemolysis ratio of the surface by 53%, compared with unmodified PU.

### 3.10. In-Vivo Thrombogenicity

**Figure 7:**
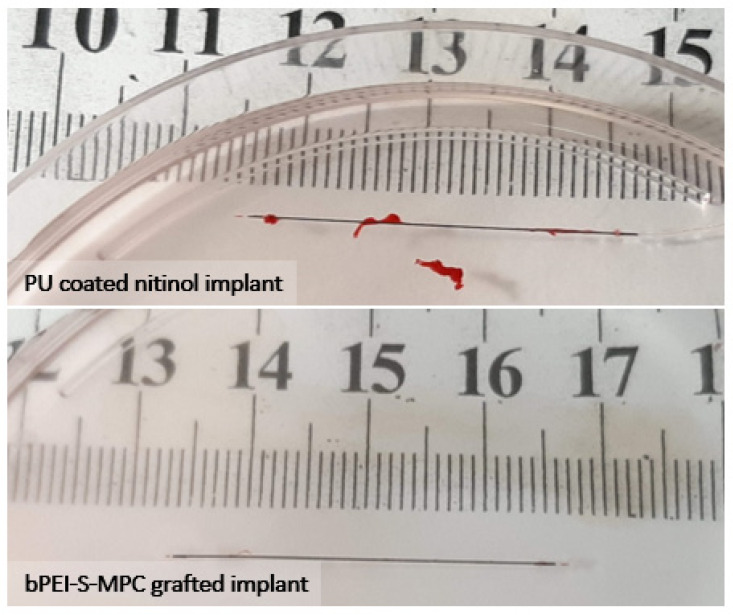
Implants after 3 h implantation in rabbit femoral artery. Implants that were grafted with bPEI-S-MPC showed significant antithrombogenic potential (average score 1.3, *n* = 3) compared with a PU-coated implant (average score 3.3, *n* = 3).

The antithrombogenic performance in-vivo of bPEI-S-MPC was evaluated using PU-coated Nitinol wires. The bPEI-S-MPC was applied to PU-coated wires to simulate its application on a finished product. Unmodified wires were used as the control group (Supplementary Figure S13). The in vivo assay was based on the Wessler stasis model in rabbits. Three hours after implantation in the femoral artery, the samples were removed and microscopically observed (Supplementary Figure S14). The thrombus formed was given a clot severity score from 0 to 4. As shown in Figure 7, implants that were grafted with bPEI-S-MPC showed significant antithrombogenic potential (average score 1.3) with *n* = 3, compared with the PU-coated implant (average score of 3.3).

## 4. Conclusions

In this study, a grafting method was developed to be grafted onto industrial PU catheters. Compared to the neat PU, bPEI-S-MPC-grafted PU exhibited antithrombogenic and antimicrobial activity, alongside reduced friction and a reduced hemolytic ratio. A covalent bond between the bPEI-S-MPC complex and the PU surface was mediated by a diisocyanate molecule. The grafting method presented here may help to maintain the structure and formulation of existing PU catheters, as it could be applied to commercial devices in a relative simple manner via a straightforward dip-coating technique, allowing high throughput and scalability of the process.

## Figures and Tables

**Table 1:** Atomic content in the coating complex, obtained by elemental analysis.

Atomic%	bPEI	bPEI-SH	bPEI-S-MPC
**C**	51.2	52.3	48.9
**H**	11.1	8.3	10.3
**N**	32.7	29.6	21.9
**S**	0	2.6	2.1
**O**	2.3	3.2	10.3
**P**	0	0	1.62

**Table 2:** Atomic content of PU-HDI-bPEI detected by XPS analysis.

	Atomic content (%)			Atomic Ratio	
	**C**	**N**	**O**	**O/N**	**N/C**
**PU neat**	71.49	2.05	50.17	24.47	0.03
**PU-HDI**	69.61	12.07	15.64	1.3	0.17
**PU-HDI-bPEI**	71.29	8.69	18.28	2.1	0.12

**Table 3:** CFU count on blood-agar Petri dishes after JIS Z2801:200 test for antimicrobial activity.

	CFU
**PU**	˃200
**PU-HDI**	˃200
**PU-HDI-bPEI**	~50
**PU-HDI-bPEI-SH**	0
**PU-HDI-bPEI-MPC**	0

## Supplementary Materials

The following are available online at https://www.mdpi.com/2073-4360/12/5/1131/s1, Figure S1: Figure S1. COF measurement apparatus, Figure S2: ATR-FTIR spectra of PU-TSC, PU neat and TSC reagent. Figure S3: Close up of the detection of sulfonyl group on modified PU surface (PU-TSC). Figure S4: Comparison of catalyzed (PU-TSC) and uncatalized (Control – No cat) functionalization of PU surface at: 70 ˚C for 60 min (upper curves) and room temperature for overnight (lower curves). Figure S5. ATR-FTIR spectra of the functionalization of PU surface with TSC (PU-TSC) at RT for: 30; 45; 60; 120 min. Figure S6: ATR-FTIR spectra of the functionalization of PU surface with TSC (PU-TSC) for 60 min at: room temperature; 70 ˚C and ice cold water. Figure S7. Optimization of the time and temperature of the functionalization of PU with TSC using efficiency factors A1157/A1527. Figure S8. Elastic modulus of PU neat; functionalized PU surface (PU-NCO) and PU which was treated with the reaction conditions (solvent, time and temperature) (PU treated), obtained by tensile test. Figure S8: Elastic modulus of PU neat; functionalized PU surface (PU-NCO) and PU which was treated with the reaction conditions (solvent, time and temperature) (PU treated), obtained by tensile test. Figure S9: Strength of PU neat; functionalized PU surface (PU-NCO) and PU which was treated with the reaction conditions (solvent, time and temperature) (PU treated), obtained by tensile test. Figure S10. Elongation at break of PU neat; functionalized PU surface (PU-NCO) and PU which was treated with the reaction conditions (solvent, time and temperature) (PU treated), obtained by tensile test. Figure S11: The evolution steps of the coating procedure: PU; PU-HDI; PU-HDI-bPEI; PU-HDI-bPEI-SH; PU-HDI-bPEI-S-MPC. Figure S12: Blood-agar petri dishes after JIS Z2801:200 test for antimicrobial activity. Figure S13: The exposure of the Femoral artery; the implant before insertion. Figure S14. Microscopical observation of the tested implants after 3 hours of implantation. Table S1: XPS analysis of PU-TSC compared to unmodified PU surface. Table S2: Series of reactions for the optimization of the reaction between isocyanate and urethane linkage. Scheme S1: The reaction scheme between urethane and isocyanate, resulting in the formation of an allophanate bond. Scheme S2: The reaction between toluenesulfonyl isocyanate (TSC) and urethane linkage.

## Abbreviations

bPEI	Branched polyethyleneimine
bPEI-SH	Thiol terminated branched polyethyleneimine (thiolated bPEI)
bPEI-S-MPC	MPC terminated branched polyethyleneimine, mediated by ethylene sulfide. Also called the grafting complex
PU-HDI	Hexamethylene diamine (HDI) grafted on PU surface
PU-HDI-bPEI	bPEI terminated PU surface, mediated by HDI
PU-HDI-bPEI-SH	bPEI-SH terminated PU surface, mediated by HDI
PU-HDI-bPEI-S-MPC	bPEI-S-MPC terminated PU surface, mediated by HDI. Also referred as the end product
