# Supplementary material for: The Grafting of Multifunctional Antithrombogenic Chemical Networks on Polyurethane Intravascular Catheters
Source: Polymers (Basel). 2020 May 15;12(5):1131. doi: 10.3390/polym12051131 (PMC7284597; doi:10.3390/polym12051131)
Supplement: Supplementary file 1 [file polymers-12-01131-s001.pdf]

Electronic supplementary information

The grafting of multifunctional antithrombogenic chemical networks on polyurethane intravascular catheters

Yael Roth, Dan Y. Lewitus

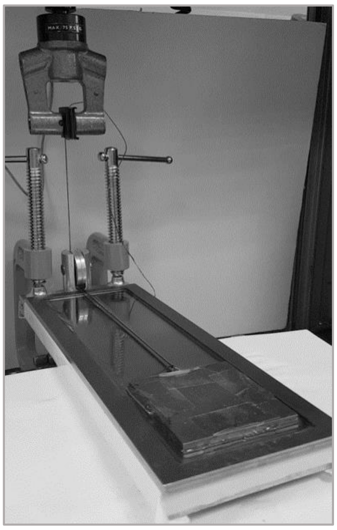

Figure **S1.** COF measurement apparatus.

Optimization of PU Functionalization using toluenesulfonyl isocyanate

PU-TSC was synthesized as follows: PU surface was soaked in toluene with 0.25% v/v of DBTDL. The reaction was conducted for 30, 45, 60, 120 min or overnight at three different temperatures (RT, 70 ˚C or ice water) under orbital shaking at 50 rpm. The effect of the reaction conditions was evaluated using ATR-FTIR spectrophotometer and elemental analysis.

The reaction between isocyanate functional group and the secondary nitrogen, which is present in urethane linkage results in the formation of a substituted urea linkage, also called allophanate, as shown in scheme S1. Toluenesulfonyl isocyanate (TSC) is a monofunctional substance used to simplify the optimization of the reaction conditions. The presence of sulfonyl group facilitates the analysis of the resulting products. The reaction between TSC and PU (Scheme S2) was monitored using ATR-FTIR and XPS.

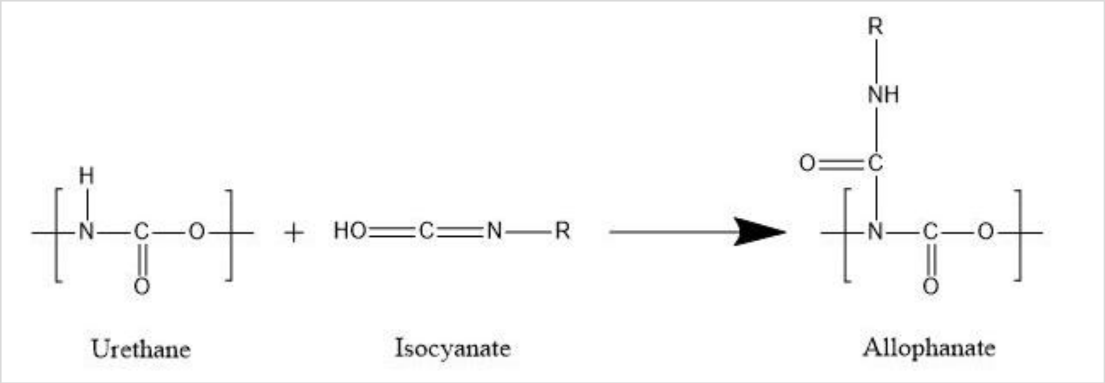

Scheme S1. The reaction scheme between urethane and isocyanate, resulting in the formation of an allophanate bond.

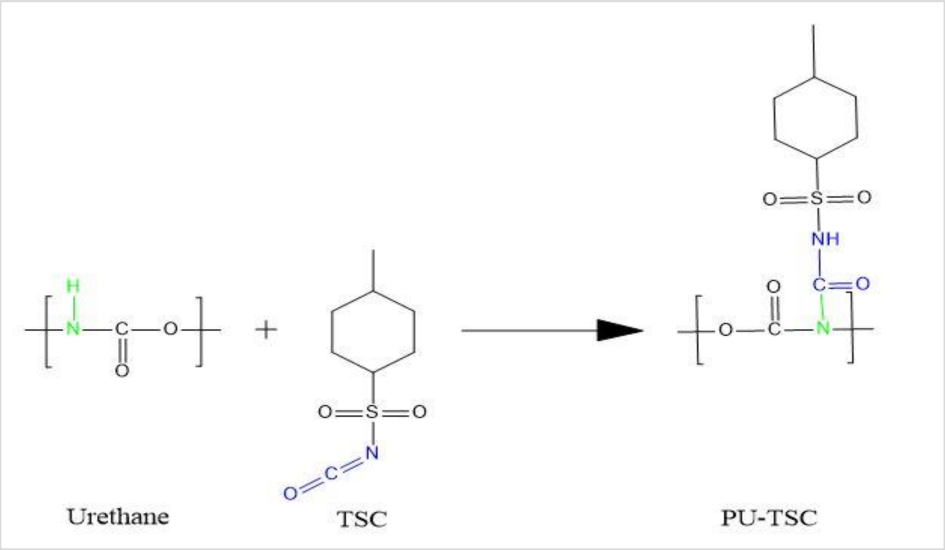

Scheme S2. The reaction between toluenesulfonyl isocyanate (TSC) and urethane linkage.

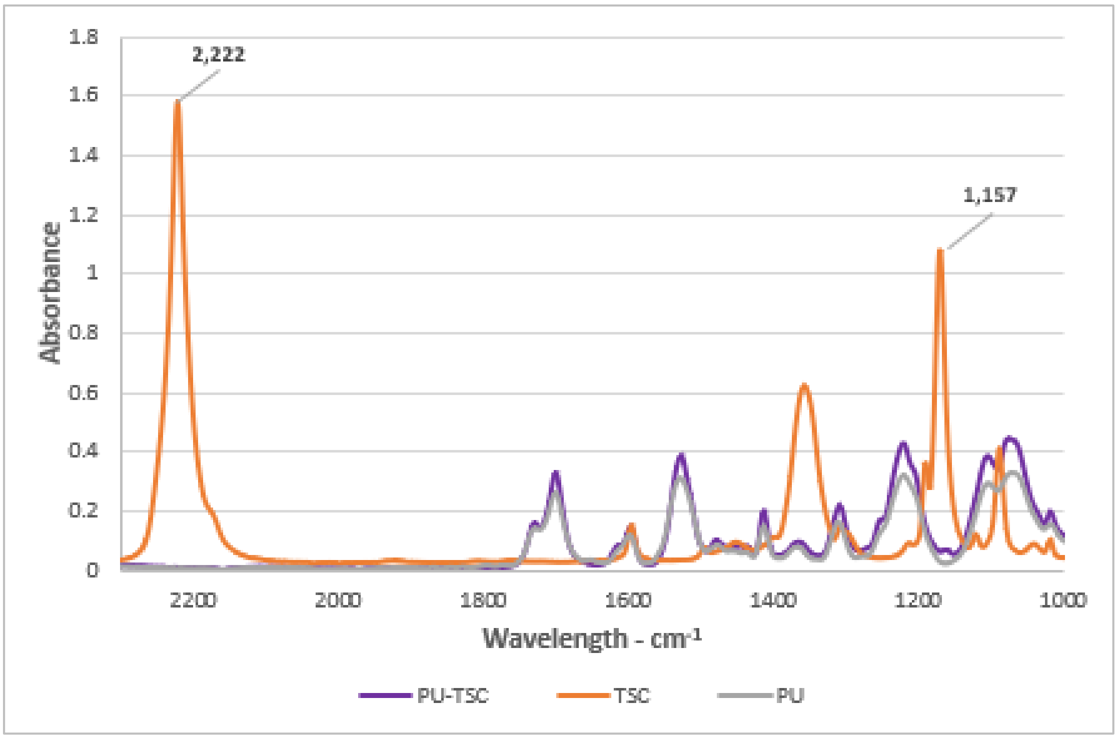

Figure S2. ATR-FTIR spectra of PU-TSC, PU neat and TSC reagent.

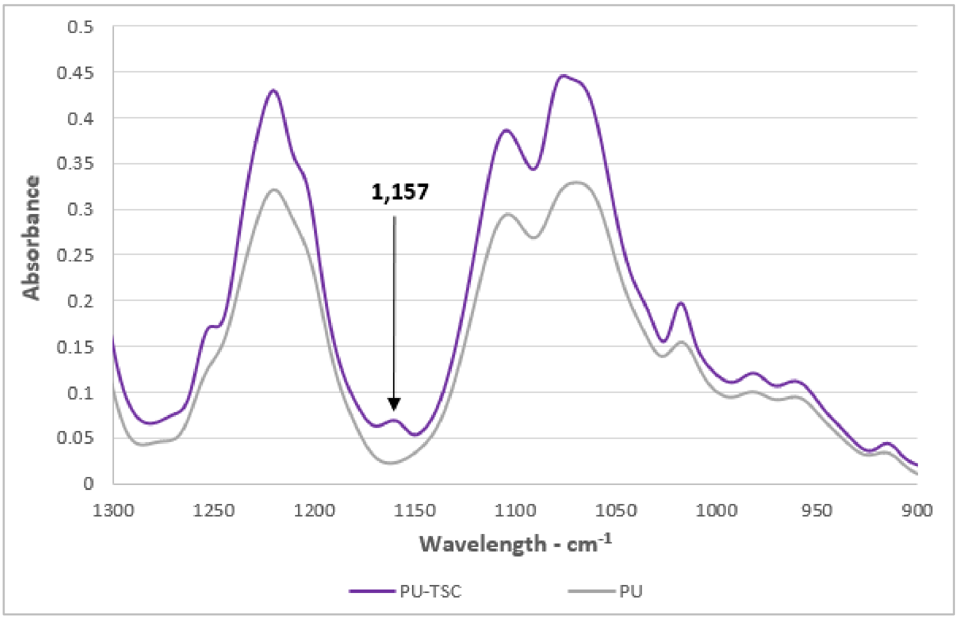

Figure S3. Close up of the detection of sulfonyl group on modified PU surface (PU-TSC).

Figure S2 shows ATR-FTIR spectra of PU, TSC and PU-TSC. The peak at 1157 cm^{-1} stands for the absorption of the sulfonyl group. This peak could be found on the functionalized PU surface (magnification of the relevant area is shown in Figure S3), indicating the presence of sulfonyl groups after the treatment. Moreover, the peak at 2222 cm^{-1}, which represents the isocyanate group could not be detected on the functionalized PU surface. These two findings indicates for the binding of TSC on PU

surfaces. The results of XPS analysis, which are given in Table S1, confirms the presence of sulfur atoms on the functionalized PU surfaces.

Table S1. XPS analysis of PU-TSC compared to unmodified PU surface.

**Atomic %**	**PU-ref**	**PU-TSC**
C	73.72	69.88
N	1.58	4.41
S	0	1.47
O	22.08	22.91

Optimization of the reaction between isocyanate and urethane linkage

A series of functionalization reactions were made (Table S2) to evaluate several procedure parameters, including the reaction temperature, duration and the necessity of catalyst within the reaction. All the reactions conducted on a flat, 200 gr PU sample in 2 ml of toluene as the solvent.

Table S2. Series of reactions for the optimization of the reaction between isocyanate and urethane linkage.

TSC [ml]	Catalyst [ml]	Reaction time [min]	Temperature [C ˚]
0.2	5	Over night	RT
0.2	0	Over night	RT
0	5	Over night	RT
0.2	5	30	RT
0.2	5	45	RT
0.2	5	60	RT
0.2	5	120	RT
0.2	5	60	70
0.2	0	60	70
0	5	60	70
0.2	5	45	70
0.2	5	60	Ice water

Figure S4 shows the formation of sulfonyl group on uncatalyzed surface (gray curve), indicating that the reaction can occur without a catalyst. The same tendency is shown at room temperature reaction for overnight, as well as at 70 ˚C for 60 min. However,

without catalyst the intensity of the sulfonyl peak is significantly weaker in comparison to the catalyzed reaction. Therefore, the catalyst is thought as necessary in the development of an industrial procedure, where the efficiency of the reaction is highly important.

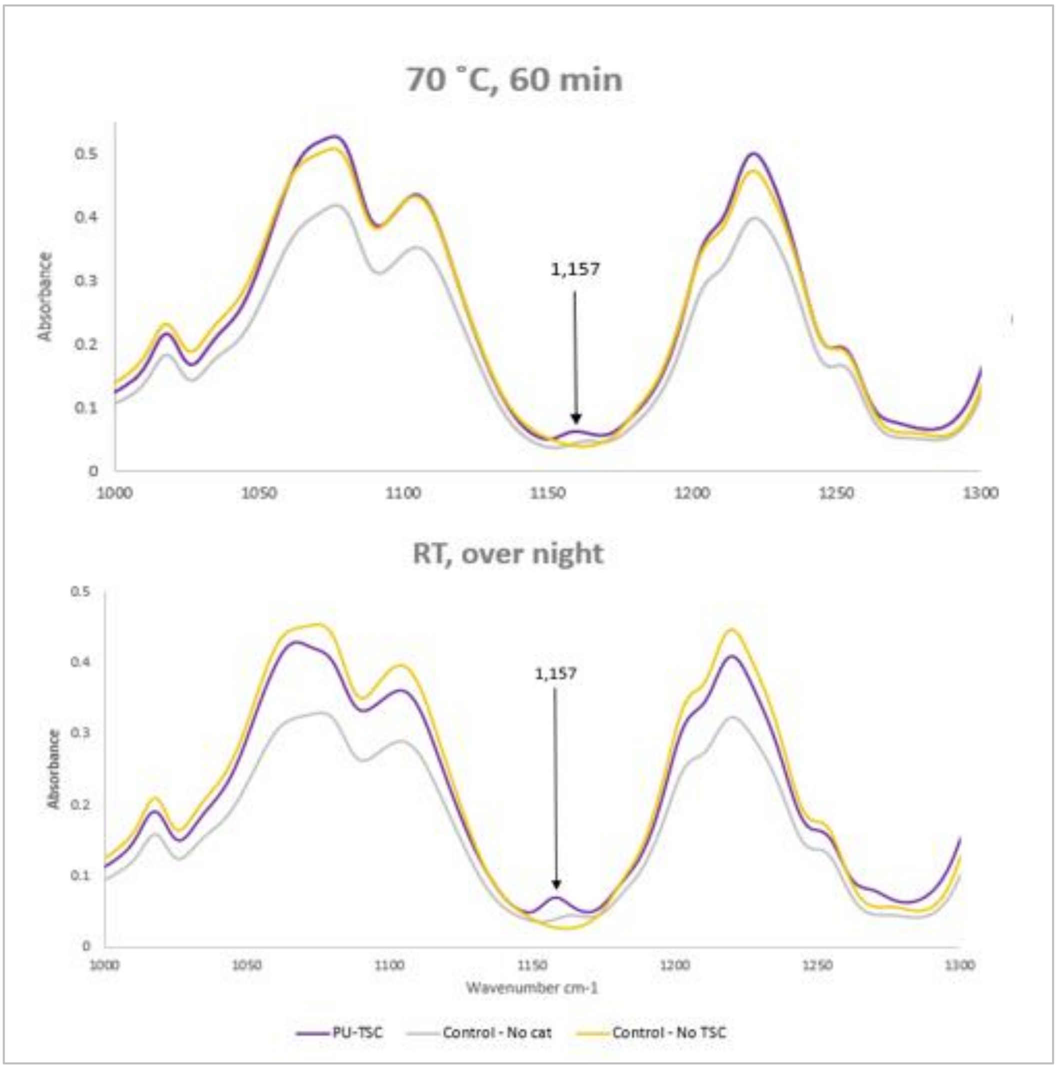

Figure S4. Comparison of catalyzed (PU-TSC) and uncatalized (Control – No cat) functionalization of PU surface at: 70 ˚C for 60 min (upper curves) and room temperature for overnight (lower curves).

The reaction was also conducted at 30, 45, 60 and 120 min at RT, 70 ˚C and in ice water. Figure 5 and 6 shows the ATR-FTIR absorbance for the reaction in different durations and temperatures, respectively. A semi-quantifying analysis was made by peak integration. As the efficiency factor, the peak area of the sulfonyl group at 1157 cm^{-1} (A1157) was divided by the peak area of a reference absorption peak of PU at 1527 cm^{-1} (A1527). The efficiency of the reaction parameters is summarized in Figure

S7. As the temperature of the reaction increases, more sulfonyl groups were generated on the surface. Additionally, the efficiency of the reaction was increased with time, reaching a maximum at 60 min. The optimization of the reaction conditions shows that the ideal product is observed at a temperature of 70˚C for 60 min.

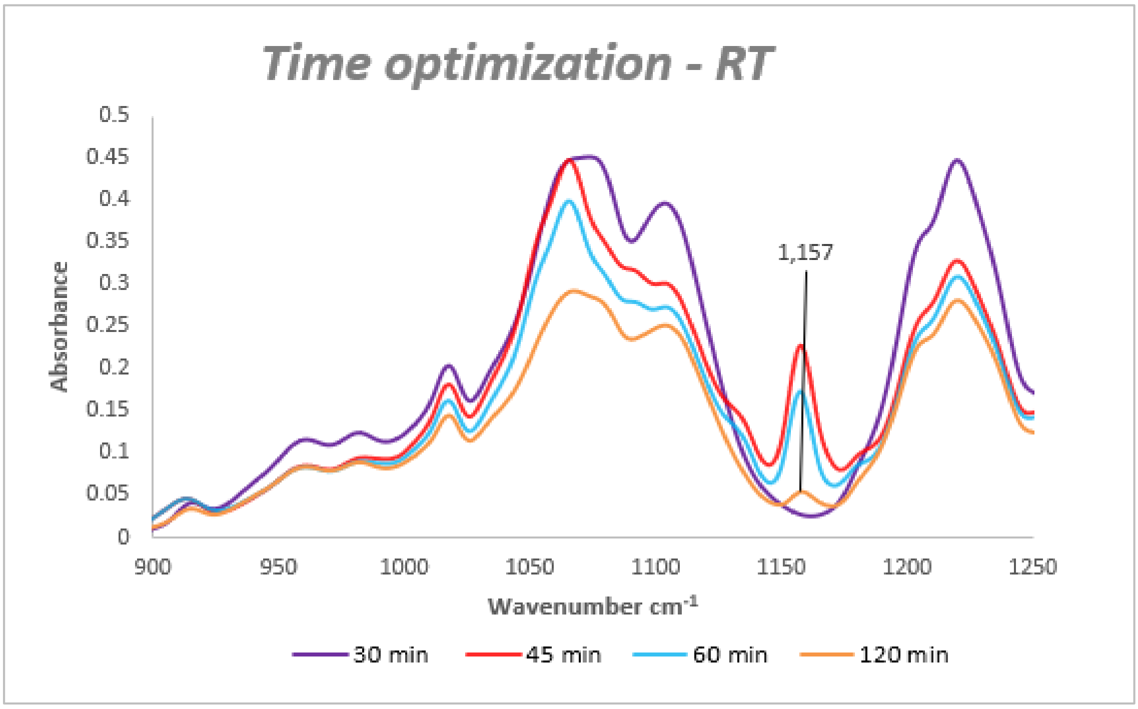

Figure S5. ATR-FTIR spectra of the functionalization of PU surface with TSC (PU-TSC) at RT for: 30; 45; 60; 120 min.

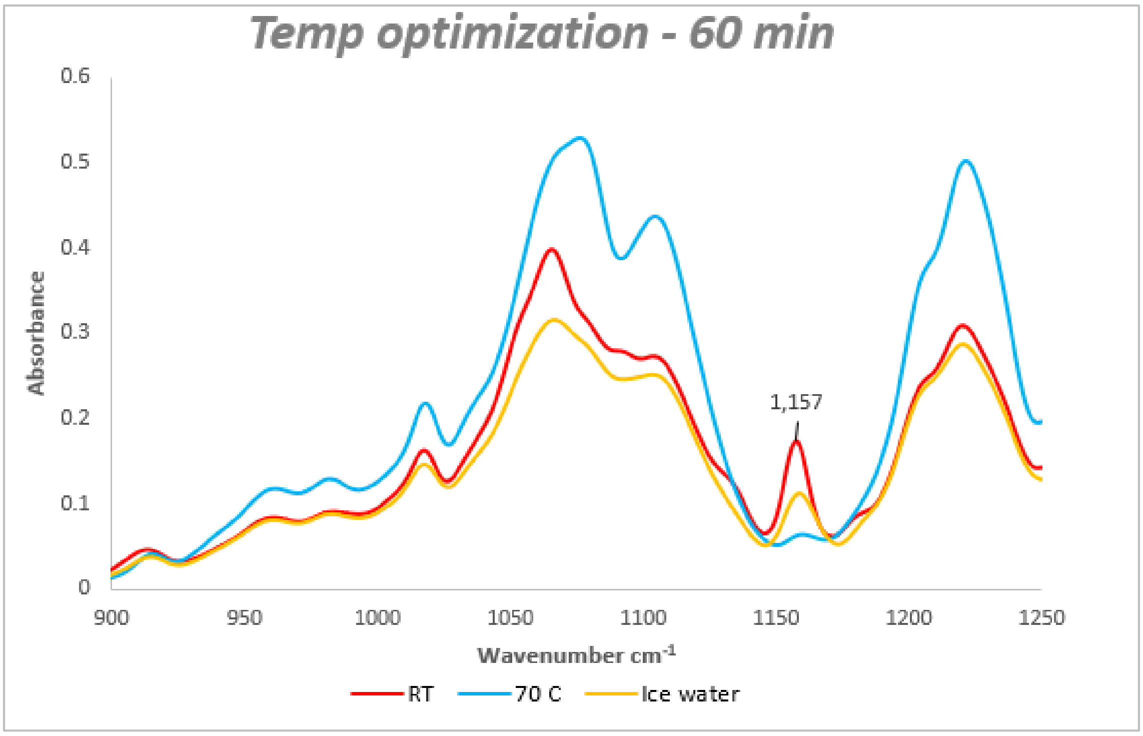

Figure S6. ATR-FTIR spectra of the functionalization of PU surface with TSC (PU-TSC) for 60 min at: room temperature; 70 ˚C and ice cold water.

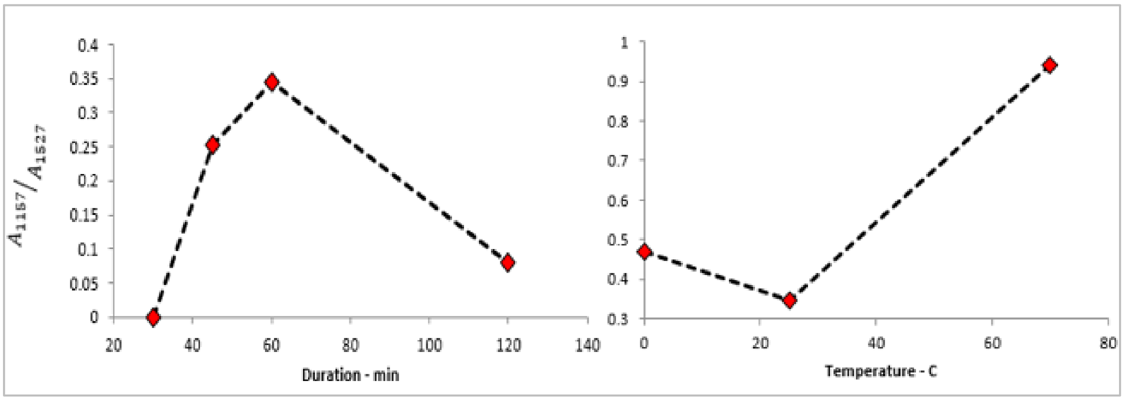

Figure S7. Optimization of the time and temperature of the functionalization of PU with TSC using efficiency factors A1157/A1527.

Mechanical analysis

Tensile tests performed in order to evaluate whether the process affect the inherent mechanical properties of the PU. Test were performed according to ASTM D638, using an Instron® 4481 tensile tester with a 100N load cell. Coated and uncoated PU samples films were cut using a standard puncher to a size of 3 X 63 mm^2. The samples tested were solvent cast PU, PU that went through the reaction conditions including solvent, catalyst and temperature (PU treated) and the functionalized PU-HDI.

Mechanical properties

The grafting of HDI on the surface of PU, as well as the exposure of the substrate to the reaction conditions, can potentially influence the mechanical properties of PU. Figures S8, S9 and S10 exhibit the elastic modulus, tensile strength and elongation at break of the samples, respectively. High standard deviations probably result from an uneven evaporation of THF after solvent casting. However, there was no significant influence of the reaction conditions or the grafting of HDI on the mechanical properties of the substrates.

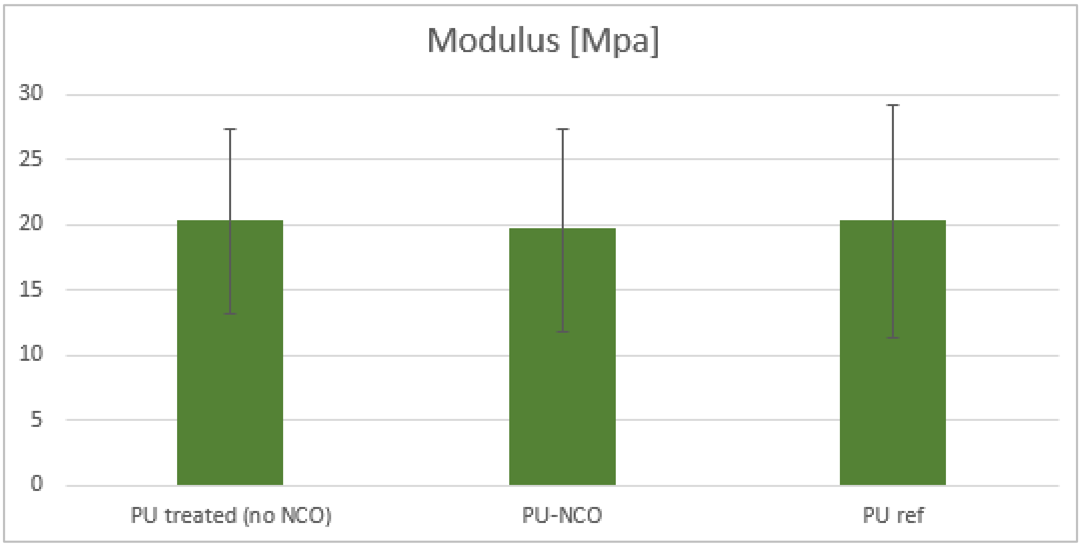

Figure S8. Elastic modulus of PU neat; functionalized PU surface (PU-NCO) and PU which was treated with the reaction conditions (solvent, time and temperature) (PU treated), obtained by tensile test.

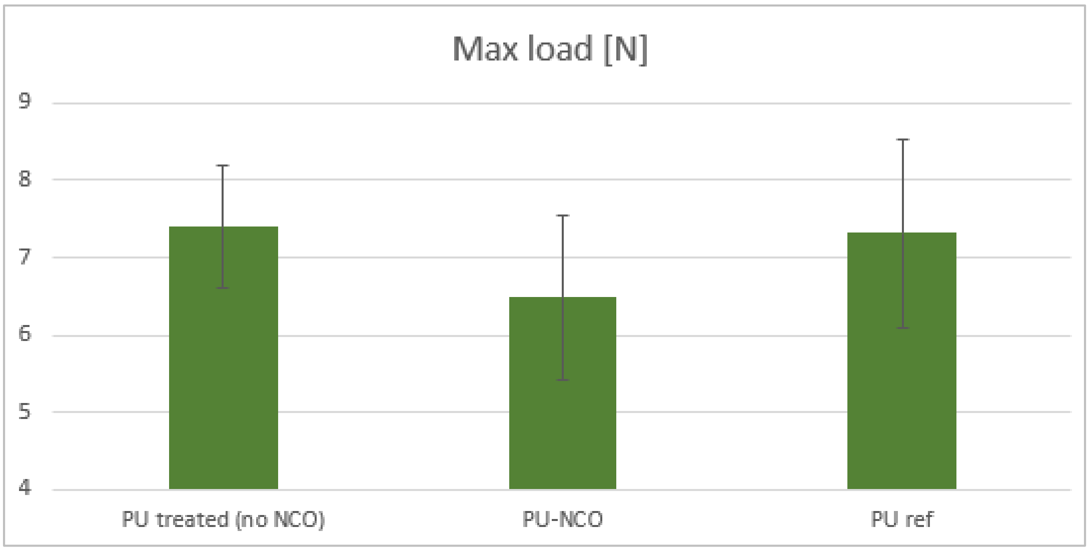

Figure S9. Strength of PU neat; functionalized PU surface (PU-NCO) and PU which was treated with the reaction conditions (solvent, time and temperature) (PU treated), obtained by tensile test.

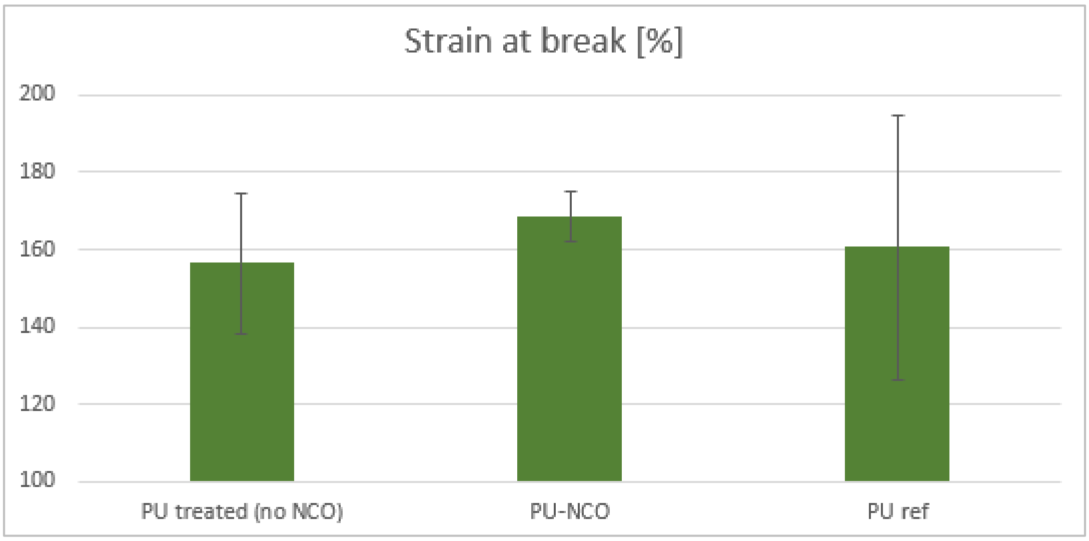

Figure S10. Elongation at break of PU neat; functionalized PU surface (PU-NCO) and PU which was treated with the reaction conditions (solvent, time and temperature) (PU treated), obtained by tensile test.

Ex-vivo evaluations

Anti-microbial activity

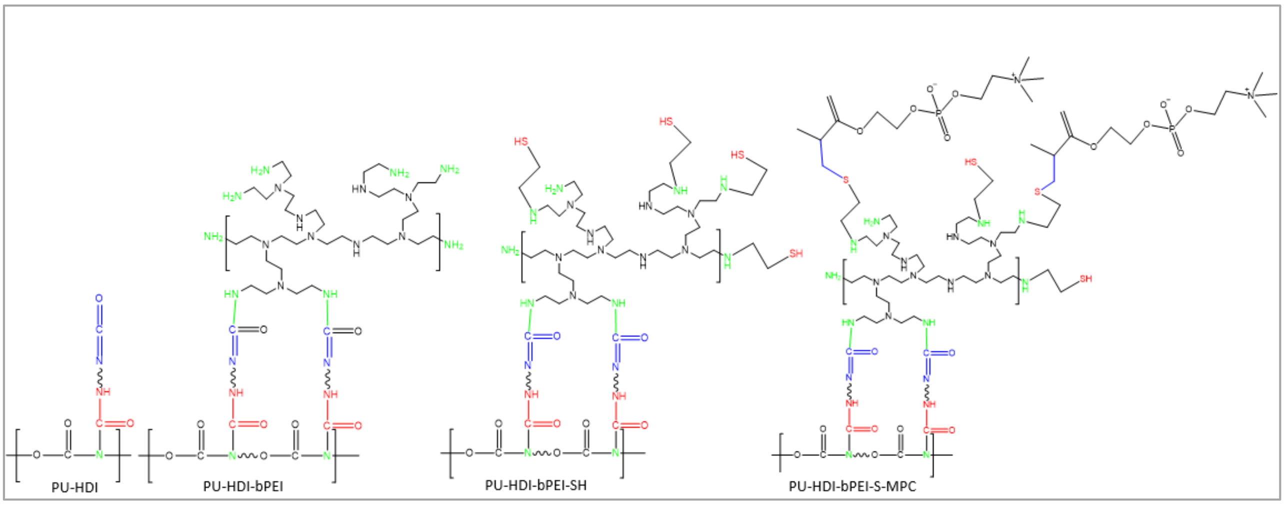

Figure S11. The evolution steps of the coating procedure: PU; PU-HDI; PU-HDI-bPEI; PU-HDI-bPEI-SH; PU-HDI-bPEI-S-MPC.

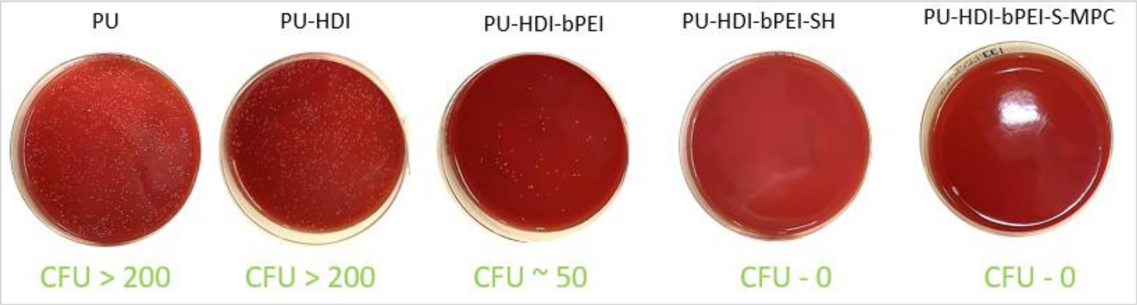

Figure S12. Blood-agar petri dishes after JIS Z2801:200 test for antimicrobial activity.

In-vivo evaluations

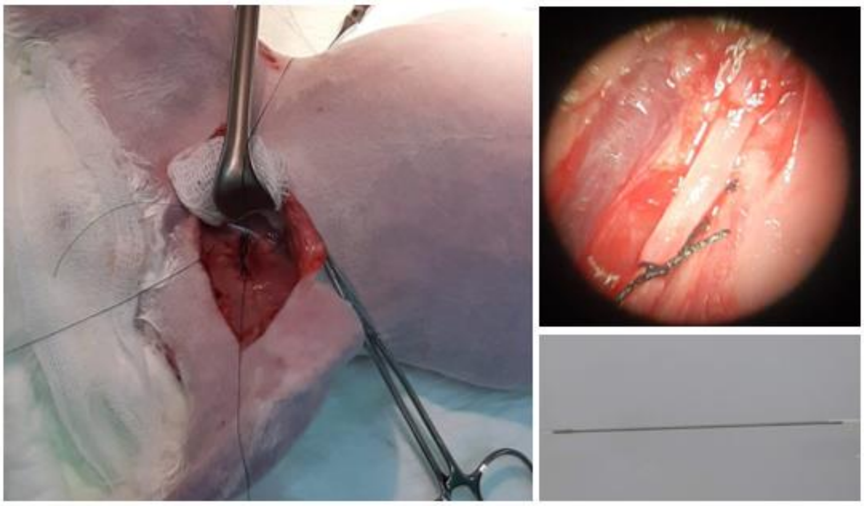

Figure S13. The exposure of the Femoral artery; the implant before insertion.

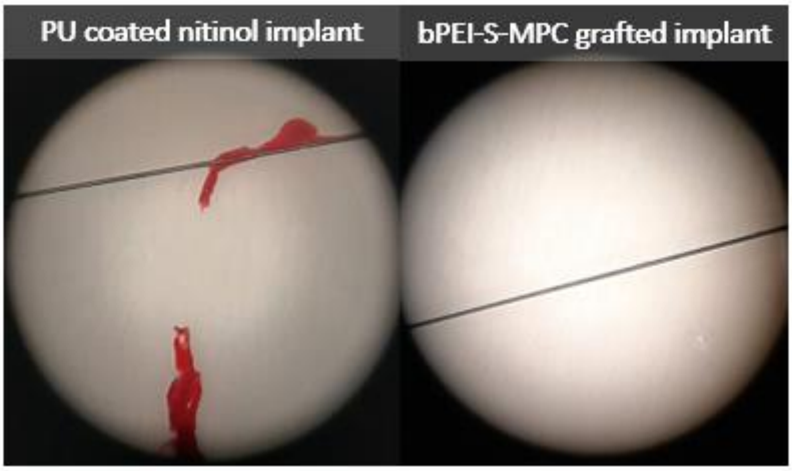

Figure S14. Microscopical observation of the tested implants after 3 hours of implantation.
